# Axial and Shear Buckling Analysis of Multiscale FGM Carbon Nanotube Plates Using the MTSDT Model: A Numerical Approach

**DOI:** 10.3390/ma15072401

**Published:** 2022-03-24

**Authors:** Ravi Kumar, Ajay Kumar, Małgorzata Szafraniec, Danuta Barnat-Hunek, Joanna Styczeń

**Affiliations:** 1 Department of Civil Engineering, National Institutes of Technology Patna, Patna 800005, India; raviroy606@gmail.com; 2 Faculty of Civil Engineering and Architecture, Lublin University of Technology, Nadbystrzycka 40, 20-618 Lublin, Poland; d.barnat-hunek@pollub.pl (D.B.-H.); j.styczen@pollub.pl (J.S.)

**Keywords:** axial and shear buckling analysis, modified third-order shear deformation theory, finite element model, carbon nanotube, Halpin–Tsai equation

## Abstract

The present paper investigates the axial and shear buckling analysis of a carbon nanotube (CNT)-reinforced multiscale functionally graded material (FGM) plate. Modified third-order deformation theory (MTSDT) with transverse displacement variation is used. CNT materials are assumed to be uniformly distributed, and ceramic fibers are graded according to a power-law distribution of the volume fraction of the constituents. The effective material properties are obtained using the Halpin–Tsai equation and Voigt rule of the mixture approach. A MATLAB code is developed using nine noded iso-parametric elements containing 13 nodal unknowns at each node. The shear correction factor is eliminated in the present model, and top and bottom transverse shear stresses are imposed null to derive higher-order unknowns. Comparisons of the present results with those available in the literature confirm the accuracy of the existing model. The effects of material components, plate sizes, loading types, and boundary conditions on the critical buckling load are investigated. For the first time, the critical buckling loads of CNT-reinforced multiscale FGM rectangular plates with diverse boundary conditions are given, and they can be used as future references.

## 1. Introduction

In the analysis and design of all civil engineering structures, the buckling response of the CNT-reinforced FGM plate caught the attention of many researchers in recent years. Currently, critical buckling loads are obtained using the Corr and Jennings [1] simultaneous iteration technique. The critical buckling load is the maximum load in the elastic range of the material above which plates start to deflect laterally. If the material is stressed beyond the elastic range and into the non-linear (plastic) range, the buckling strength of a plate is smaller than the elastic buckling strength of a plate. When the load approaches the critical buckling load, the plate will bend significantly, and the material’s stress–strain behavior will diverge from linear. In FGM-type composite material, properties of material constituents are varied according to the required performance. In this paper, the material constituents were a metal matrix, CNT reinforcement, and fiber. The final material was made in two phases. Here, we calculated the minimum edge compressive load in the form of the non-dimensional critical buckling load, which is required to initiate the instability of the plate structure. FGM is widely employed in many areas such as machine, construction, defense, electronic, chemical, pharma, energy sources, nuclear, automotive, and shipbuilding industries. Because of the expanding use of FGMs in a range of structural applications, detailed theoretical models are required to anticipate their behavior.

Abrate [2] used a classical plate theory, FSDT, and HSDT to study the dynamic, static, and buckling behaviors of thick and thin FGM plates. The significance of their study is that the response of the FGM plate can be analyzed without performing a direct analysis. Zenkour [3] adopted a generalized shear deformation model to study the stress and displacement of FGM plates under uniform loading. They observed that the gradient material properties play a vital role in the response of the FGM plates. Zhang [4] carried out a geometric non-linear analysis of CNT-reinforced FGM plates with column support. For modeling the structure, they used FSDT mathematical model with the von Kármán nonlinearity equation. Based on HSDT theory the Levy-type solution has been presented by Bodaghi andSaidi [5] for buckling analysis of simply supported FGM plate to observe the effect of the various parameter such as volume fraction index, aspect ratio, side-thickness ratio, loading condition, and various boundary condition. Thai and Choi [6] developed a refined displacement theory without considering the shear correction factor for calculating the critical buckling load of the FGM plates. Various numerical studies have been presented for dynamic, buckling, and post-buckling analysis of FGM plate, laminated, and shell structure [7,8,9,10,11,12,13].

Kiani [14] studied the buckling response of a CNT-based FGM plate subjected to mechanical load. The distribution of load is obtained using the 2D formulation. Feldman and Aboudi [15] studied the buckling behavior of uniaxially loaded FGM plates. A combination of micromechanical and structural approaches is used to predict the effective material properties of non-homogeneous FGM plates. Zghal et al. [16] carried out the buckling response of FGM- and CNT-reinforced FGM plates and cylindrical panels. The final material properties of these plates and cylindrical panels were achieved by the power law and the extended rule of a mixture. A simple power-law equation for calculating the effective material properties was used by Ramu and Mohanty [17] for buckling analysis of FGM plates using the FEM method and noting that the critical buckling load in non-axial compression was greater than that in biaxial compression. Arani et al. [18] used an analytical and a finite element approach to determine the critical buckling load of the CNT-reinforced composite plate, and the overall elastic properties of the material were calculated by the Mori–Tanaka approach. By adopting the simple rule of a mixture, the effective elastic properties of the FGM sandwich were calculated by Yaghoobi and Yaghoobi [19] to calculate the critical buckling load under mechanical, thermal, and thermo–mechanical loading. A micromechanics model based on Halpin–Tsai and the extended mixture rule has been used by Hanifehlou and Mohammadimehr [20] to predict the effective elastic properties of graphene platelets and CNT-reinforced FGM plates. Lei et al. [21] and Wang et al. [22] considered an extended rule of mixture approach for predicting the effective material properties of CNT-reinforced FGM for buckling analysis. By assuming the power law composition of the volume fraction of the constituent material, the effective material properties were calculated to investigate the buckling analysis of the FGM plate structure [23]. Aragh et al. [24] employed the Eshelby–Mori–Tanaka method to calculate the effective elastic properties of the material for vibration response of a continuous-grade CNT-reinforced cylindrical panel.

Bouguenina et al. [25] presented a solution to investigate the thermal buckling analysis of FGM plates. The presented solution was based on an analytical approach for constant thickness and a finite element approach for variable thickness. Mirzaei and Kiani [26] studied the thermal buckling analysis of CNT-reinforced FGM plates, where CNT and the matrix material were assumed to be temperature-dependent. Singh et al. [27] studied the buckling and vibration analysis of isotropic and sandwich FGM plates resting on an elastic foundation. They adopted a new sigmoid law to predict the effective elastic properties of the FGM plate. The buckling response and post-buckling response of pristine composite plates reinforced with graphene sheets were investigated by Zeverdejani et al. [28]. The stability equations were solved using the eigenvalue problem, and the critical buckling loads were calculated for various boundary conditions. Fekrar et al. [29] studied the buckling analysis of a ceramic-based FGM plate using only four-variable refined theory and demonstrated the accuracy and effectiveness of mathematical theory in analyzing the buckling behavior. A refined plate theory based on the secant function was used by Abdulrazzaq et al. [30] to study the thermal buckling stability of clamped nano-size FGM plates. From their study, it can be observed that the buckling behavior of clamped FGM nanoplates was very sensitive to various parameters such as aspect and side-to-thickness ratios, material graduation, thermal condition, etc. The study of the influence of small-scale parameters on the vibration and buckling behavior of CNT-reinforced FGM plates was done by Shahraki et al. [31]. The CNT-based FGM nanoplate was considered to rest on a Kerr elastic foundation. Costa and Loja [32] represented the static analysis of a dual-phase moderately thick FGM plate. The CNT reinforcements were assumed to be added to the matrix material in the first phase.

Even though various studies on the buckling of FGM plates have been conducted based on a range of plate theories, no studies on the buckling of multiscale FGM plates based on the MTSDT theory were found. The present MTSDT mathematical theory has been modified to represent the kinematics field that captures normal and transverse cross-section deformation modes. The assumed in-plane fields incorporate the cubic degree of thickness terms and quadratic degree of thickness terms for the transverse component. The C1 continuity requirement associated with third-order shear deformation theory is avoided by developing a C0 FE formulation by replacing the out-of-plane derivatives with independent field variables. The present study can be used for the design and analysis of various types of hybrid composite curve panels, which are used in various engineering fields. The design charts can be obtained by the present model, which may be useful for the designer. Material properties, such as Young’s modulus, are supposed to change with plate thickness according to a power-law distribution of the volume percentage of the constituents. To the best of the authors’ knowledge, no experimental results on the present work are available in the literature; hence, present model results were validated with the closed-form elasticity solution and numerical analysis results available in the literature. To study the influence of various parameters, the non-dimensional critical buckling load was calculated for numerical analysis.

## 2. Geometrical Configuration and Effective Material Properties

**Figure 1:**
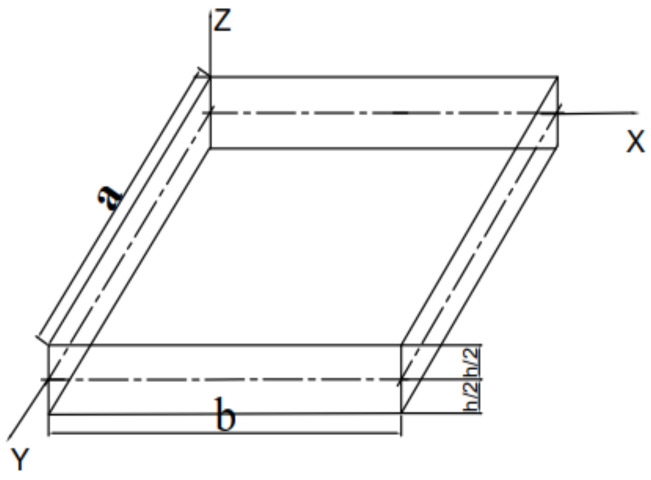
Geometrical configuration of the plate.

A multiscale FGM plate of length a, width b, and thickness h, as shown in Figure 1 was considered. In the buckling response of the plate, the rectangular Cartesian platform coordinates × and y were used. The co-ordinate planes × = 0, a and y = 0, b define the boundaries of the plate. The reference surface is the middle surface of the plate, defined by z = 0, where z is the thickness co-ordinate measured from the un-deformed middle surface of the plate.

**Figure 2:**
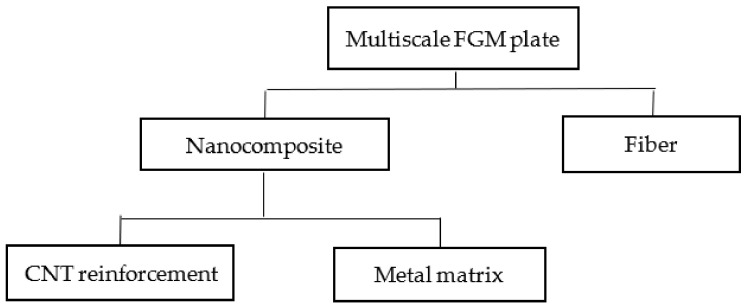
Hierarchy of the three-phase multiscale FGM plate.

The performance of these FGM plates might be improved by using a multiscale hierarchical FGM as shown in Figure 2, which is made possible by combining the continuous fiber phase, the metal matrix, and CNT reinforcement. In such circumstances, the overall homogenization process can be divided into two phases: in the first phase, the dispersion of CNT in the metallic matrix yields a nanocomposite, and in the second phase, this nanocomposite receives ceramic inclusions in a graded manner, resulting in a CNT-reinforced multiscale composite. Since the CNTs are expected to be evenly distributed and randomly oriented throughout the matrix, the final mixture is considered an isotropic mixture. It is also expected that the bonding between CNT and matrix and dispersion of CNT in the matrix are perfect. Each CNT is assumed to be straight and has the same aspect ratio and mechanical properties. The matrix material is considered void-free, and the bonding between the matrix and fiber is excellent.

To evaluate the effective elastic properties of the material, a suitable approach should be adopted. A combination of the Halpin–Tsai equation [33] and homogenization scheme can be adapted to predict the effective material properties of a three-phase multiscale FGM plate. The Halpin–Tsai equation is an empirical formula, known to be fit for calculating effective material properties of the mixture of the matrix and low fraction of the CNT reinforcement. The elastic properties of an anisotropic mixture of CNT and the matrix can be expressed as follows:

(1) $$E_{MNC}=\frac{E_{M}}{8}\left(5\left(\frac{1+2\alpha V_{CN}}{1-\alpha V_{CN}}\right)+3\left(\frac{1+2\frac{l}{d}\beta V_{CN}}{1-\beta V_{CN}}\right)\right)$$

(2) $$\alpha =\frac{\left(\frac{E_{CN}}{E_{M}}\right)-\left(\frac{d}{4t}\right)}{\left(\frac{E_{CN}}{E_{M}}\right)+\left(\frac{l}{2t}\right)}\;\;\;\;\;\;;\;\beta =\frac{\left(\frac{E_{CN}}{E_{M}}\right)-\left(\frac{d}{4t}\right)}{\left(\frac{E_{CN}}{E_{M}}\right)+\left(\frac{d}{2t}\right)}$$

The volume fraction of carbon nanotube $V_{CN}$ and Poisson’s ratio of the nanocomposite $\nu_{MNC}$ are calculated as [34].

(3) $$V_{CN}=\frac{W_{CN}}{W_{CN}+\left(\frac{\rho_{CN}}{\rho_{M}}\right)-\left(\frac{\rho_{CN}}{\rho_{M}}\right)W_{CN}}$$

(4) $$\nu_{MNC}=\nu_{{}_{CN}}\cdot V_{CN}+\nu_{M}\cdot (1-V_{CN})$$

The volume fraction of dispersed fiber constituents is expressed as follows:

(5) $$V_{F}={\left(\frac{z}{h}+0.5\right)}^{n}$$

where *h* and *Z* are the respective total thickness and thickness coordinate in the transverse direction, having an origin on the middle surface of the plate. The exponential power *n* permits the ceramic fiber to fluctuate in the thickness direction. The effective material characteristics of the final material fluctuate continuously according to Equation (5). In this paper, effective elastic material properties are calculated using a homogenization approach based on the Voigt rule of the mixture. as shown below:

(6) $$E(z)=(E_{C}-E_{MNC})V_{F}+E_{MNC}$$

Because of the dispersion of carbon nanotubes in the metal matrix, the effective Young’s modulus of the nanocomposite phase may be used instead of Young’s modulus of the matrix phase in the preceding equation. In this work, we assume the dispersion of carbon nanotubes in metal; therefore, we must first compute the effective material properties of the nanocomposite.

## 3. Governing Equation

The governing equation for buckling analysis is derived by using the MTSDT mathematical model. A rectangular plate of size (*a* × *b*) is assumed to be perfect in geometry.

### 3.1. Displacement Equation

The in-plane displacement (*u* and *v*) and transverse displacement (*w*), which is based on the MTSDT, are represented as follows:

(7) $$\left\{u_{i}\right\}=\left\{u_{i}^{0}\right\}+[A]f(z)$$

where $u_{i}={\left\{u,\;v,\;w\right\}}^{T},\;\;u_{i}^{0}={\left\{u_{0\;},\;v_{0},\;\;w_{0}\right\}}^{T},\;\;f(z)={\left\{z,\;z^{2},\;z^{3}\right\}}^{T}$ and

$$[A]=\left[\begin{array}{ccc} \omega_{1} & \eta_{1} & \rho_{1}\\ \omega_{2} & \eta_{2} & \rho_{2}\\ \omega_{3} & \eta_{3} & 0 \end{array}\right]$$

In the above matrix [A], all higher-order terms are determined by eliminating the transverse shear ($\tau_{xz}$ = $\tau_{yz}$ = 0) at the outer surface of the plate; then, the modified in-plane displacement field is as follows:

(8) $$\left\{\begin{array}{l} u\\ v \end{array}\right\}=\left\{\begin{array}{l} u_{0}\\ v_{0} \end{array}\right\}+f_{1}(z)\left\{\begin{array}{l} \omega_{1}\\ \omega_{2} \end{array}\right\}-\frac{z^{2}}{2}\left\{\begin{array}{l} \frac{\partial \omega_{3}}{\partial x}\\ \frac{\partial \omega_{3}}{\partial y} \end{array}\right\}-f_{2}(z)\left\{\begin{array}{l} \frac{\partial w_{0}}{\partial x}+\frac{h^{2}}{4}\frac{\partial \eta_{3}}{\partial x}\\ \frac{\partial w_{0}}{\partial y}+\frac{h^{2}}{4}\frac{\partial \eta_{3}}{\partial y} \end{array}\right\}$$

where $f_{1}(z)=z-f_{2}(z)$ and $f_{2}(z)=\frac{4z^{3}}{3h^{2}}$. The final expression for the in-plane displacement and transverse displacement fields:

(9) $$\left\{u_{i}\right\}=\left\{u_{i}^{0}\right\}+[A_{1}]f(z)$$

where

$$[A_{1}]=\left[\begin{array}{ccc} \omega_{1} & -\frac{\alpha_{1}}{2} & -\frac{4}{3h^{2}}\left(\omega_{1}+\beta_{1}+\frac{h^{2}\psi_{1}}{4}\right)\\ \omega_{2} & -\frac{\alpha_{2}}{2} & -\frac{4}{3h^{2}}\left(\omega_{2}+\beta_{2}+\frac{h^{2}\psi_{2}}{4}\right)\\ \omega_{3} & \eta_{3} & 0 \end{array}\right]$$

To replace the C^1^ continuity with C^0^ continuity to assure the field variables are continuous within the element, the out-of-plane derivatives are replaced by the following relation:

(10) $$\alpha_{1}=\frac{\partial \omega_{3}}{\partial x};\beta_{1}=\frac{\partial w_{0}}{\partial x};\psi_{1}=\frac{\partial \eta_{3}}{\partial x};\alpha_{2}=\frac{\partial \omega_{3}}{\partial y};\beta_{2}=\frac{\partial w_{0}}{\partial y};\psi_{2}=\frac{\partial \eta_{3}}{\partial y}$$

However, due to the above substitution, there is an introduction of additional nodal unknowns that impose extra constraints, which are enforced variationally through a penalty approach as follows:

(11) $$\begin{array}{l} \alpha_{1}-\frac{\partial \omega_{3}}{\partial x}=0;\;\;\beta_{1}-\frac{\partial w_{0}}{\partial x}=0;\;\psi_{1}-\frac{\partial \eta_{3}}{\partial x}=0;\;\\ \alpha_{2}-\frac{\partial \omega_{3}}{\partial y}=0;\;\beta_{2}-\frac{\partial w_{0}}{\partial y}=0;\;\psi_{2}-\frac{\partial \eta_{3}}{\partial y}=0 \end{array}$$

Hence, in the present formulation, the displacement variables are as follows:

(12) $${\left\{d\right\}}_{13X1}=\left\{u_{0},v_{0},w_{0},\omega_{1},\omega_{2},\omega_{3},\alpha_{1},\alpha_{2},\eta_{3},\beta_{1},\beta_{2},\psi_{1},\psi_{2}\right\}$$

### 3.2. Strain Displacement Relationship

The linear strain corresponding to the displacement fields is expressed as follows:

(13) $$\left\{\begin{array}{l} \varepsilon_{xx}\\ \varepsilon_{yy}\\ \varepsilon_{zz}\\ \gamma_{xy}\\ \gamma_{xz}\\ \gamma_{yz} \end{array}\right\}=\left\{\begin{array}{l} \frac{\partial u}{\partial x}\\ \frac{\partial v}{\partial y}\\ \frac{\partial w}{\partial z}\\ \frac{\partial u}{\partial y}+\frac{\partial v}{\partial x}\\ \frac{\partial u}{\partial z}+\frac{\partial w}{\partial x}\\ \frac{\partial v}{\partial z}+\frac{\partial w}{\partial y} \end{array}\right\}$$

Further incorporation of the final expression for the displacement field (Equation (9)) into the above equation leads to the following expression:

(14) $$\left\{\begin{array}{l} \varepsilon_{xx}\\ \varepsilon_{yy}\\ \varepsilon_{zz}\\ \gamma_{xy}\\ \gamma_{xz}\\ \gamma_{yz} \end{array}\right\}=\left\{\begin{array}{l} \frac{\partial u_{0}}{\partial x}\\ \frac{\partial v_{0}}{\partial x}\\ \omega_{3}\\ \frac{\partial u_{0}}{\partial y}+\frac{\partial v_{0}}{\partial x}\\ \omega_{1}+\frac{\partial w_{0}}{\partial x}\\ \omega_{2}+\frac{\partial w_{0}}{\partial y} \end{array}\right\}+z\left\{\begin{array}{l} \frac{\partial \omega_{1}}{\partial x}\\ \frac{\partial \omega_{2}}{\partial y}\\ 2\eta_{3}\\ \frac{\partial \omega_{1}}{\partial y}+\frac{\partial \omega_{2}}{\partial x}\\ \frac{\partial \omega_{3}}{\partial x}-\alpha_{1}\\ \frac{\partial \omega_{3}}{\partial y}-\alpha_{2} \end{array}\right\}+z^{2}\left\{\begin{array}{l} -\frac{1}{2}\frac{\partial \alpha_{1}}{\partial x}\\ -\frac{1}{2}\frac{\partial \alpha_{2}}{\partial y}\\ 0\\ \frac{1}{2}\left(\frac{\partial \alpha_{1}}{\partial y}+\frac{\partial \alpha_{2}}{\partial x}\right)\\ \frac{\partial \eta_{3}}{\partial x}-\psi_{1}-\frac{4}{h^{2}}(\omega_{1}+\beta_{1})\\ \frac{\partial \eta_{3}}{\partial y}-\psi_{2}-\frac{4}{h^{2}}(\omega_{2}+\beta_{2}) \end{array}\right\}+\;z^{3}\left\{\begin{array}{l} -\frac{4}{3h^{2}}\left(\frac{\partial \omega_{1}}{\partial x}+\frac{\partial \beta_{1}}{\partial x}\right)-\frac{1}{3}\frac{\partial \psi_{1}}{\partial x}\\ -\frac{4}{3h^{2}}\left(\frac{\partial \omega_{2}}{\partial y}+\frac{\partial \beta_{2}}{\partial y}\right)-\frac{1}{3}\frac{\partial \psi_{2}}{\partial y}\\ 0\\ -\frac{4}{3h^{2}}\left(\frac{\partial \omega_{1}}{\partial y}+\frac{\partial \omega_{2}}{\partial x}+\frac{\partial \beta_{1}}{\partial y}+\frac{\partial \beta_{2}}{\partial x}\right)-\frac{1}{3}\left(\frac{\partial \psi_{1}}{\partial y}+\frac{\partial \psi_{2}}{\partial x}\right)\\ 0\\ 0 \end{array}\right\}$$

The above strain equation can be generalized into the following expression:

(15) $${\left\{\bar{\varepsilon }\right\}}_{6X1}={\left\{T(z)\right\}}_{6X20}{\left\{\varepsilon \right\}}_{20X1}$$

where ${\left\{\bar{\varepsilon }\right\}}_{6X1}=\left\{\varepsilon_{xx}\;\;\varepsilon_{yy}\;\;\varepsilon_{zz}\;\;\gamma_{xy}\;\;\gamma_{xz}\;\;\gamma_{yz}\right\}$ and

$${\left\{T(z)\right\}}_{6X20}=\left[\begin{array}{cccccccccccccccccccc} 1 & 0 & 0 & 0 & 0 & 0 & z & 0 & 0 & 0 & 0 & 0 & z^{2} & 0 & 0 & 0 & 0 & z^{3} & 0 & 0\\ 0 & 1 & 0 & 0 & 0 & 0 & 0 & z & 0 & 0 & 0 & 0 & 0 & z^{2} & 0 & 0 & 0 & 0 & z^{3} & 0\\ 0 & 0 & 1 & 0 & 0 & 0 & 0 & 0 & z & 0 & 0 & 0 & 0 & 0 & 0 & 0 & 0 & 0 & 0 & 0\\ 0 & 0 & 0 & 1 & 0 & 0 & 0 & 0 & 0 & z & 0 & 0 & 0 & 0 & z^{2} & 0 & 0 & 0 & 0 & 0\\ 0 & 0 & 0 & 0 & 1 & 0 & 0 & 0 & 0 & 0 & z & 0 & 0 & 0 & 0 & z^{2} & 0 & 0 & 0 & 0\\ 0 & 0 & 0 & 0 & 0 & 1 & 0 & 0 & 0 & 0 & 0 & z & 0 & 0 & 0 & 0 & z^{2} & 0 & 0 & z^{3} \end{array}\right]$$

The relationship between the strain vector $\left\{\varepsilon \right\}$ and displacement vector $\left\{d\right\}$ can express by the following relationship:

(16) $${\left\{\varepsilon \right\}}_{13X1}={\left\{B\right\}}_{20X13}{\left\{d\right\}}_{13x1}$$

### 3.3. Element Description and Shape Function

**Figure 3:**
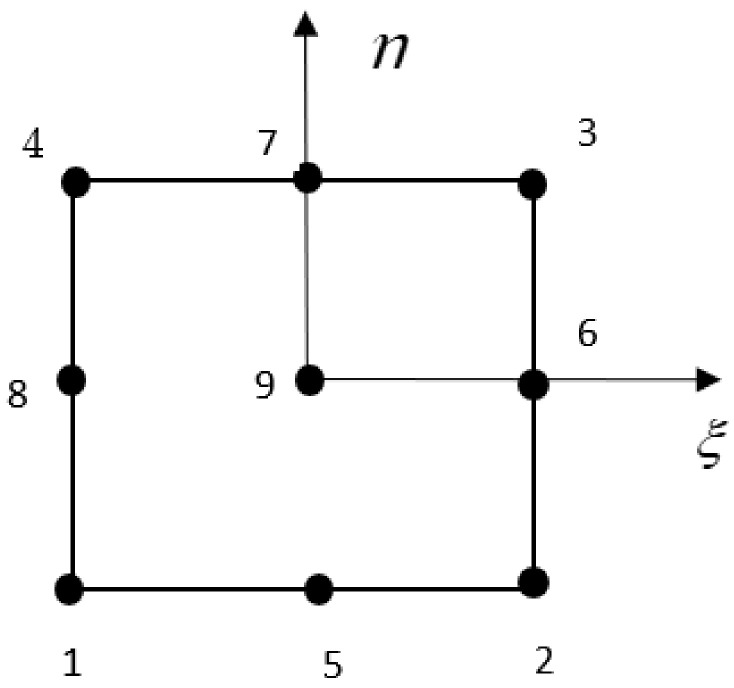
9-Noded isoparametric element.

A nine-noded iso-parametric element (shown in Figure 3) was employed for the present finite element model with 13 unknown variables at each node. The nodal unknowns at any point within the domain were expressed in terms of the shape function. At each element, the displacement field and the element geometry are defined as follows:

(17) $$\begin{array}{l} \left\{d\right\}=\sum_{i=1}^{9}N_{i}\left(\xi ,\eta \right){\left\{d\right\}}_{i}\\ \left\{x\right\}=\sum_{i=1}^{9}N_{i}\left(\xi ,\eta \right){\left\{x\right\}}_{i}\\ \left\{y\right\}=\sum_{i=1}^{9}N_{i}\left(\xi ,\eta \right){\left\{y\right\}}_{i} \end{array}$$

The shape function *N_i_* is the function of the natural coordinate system used in the finite element modeling, and it is expressed as follows:

(18) $$\begin{array}{l} N_{1}=\frac{1}{4}\left(\xi^{2}-\xi \right)\left(\eta^{2}-\eta \right),\;N_{2}=\frac{1}{2}\left(1-\xi^{2}\right)\left(\eta^{2}-\eta \right),\\ N_{3}=\frac{1}{4}\left(\xi^{2}+\xi \right)\left(\eta^{2}-\eta \right),\;N_{4}=\frac{1}{2}\left(\xi^{2}-\xi \right)\left(1-\eta^{2}\right),\\ N_{5}=\left(1-\xi^{2}\right)\left(1-\eta^{2}\right),\;\;N_{6}=\frac{1}{2}\left(\xi^{2}+\xi \right)\left(1-\eta^{2}\right),\\ N_{7}=\frac{1}{4}\left(\xi^{2}-\xi \right)\left(\eta^{2}+\eta \right),\;N_{8}=\frac{1}{2}\left(1-\xi^{2}\right)\left(\eta^{2}+\eta \right),\\ \;\;\;\;\;\;\;\;\;\;\;\;\;\;\;\;\;\;\;\;\;N_{9}=\frac{1}{4}\left(\xi^{2}+\xi \right)\left(\eta^{2}+\eta \right) \end{array}$$

### 3.4. Constitutive Relationship

In this study, we considered that the multiscale composite material is an isotropic material at each point of its domain, and the constitutive relationship between stress and strain is as follows:

(19) $$\left\{\sigma \right\}=\left[Q\right]\left\{\bar{\varepsilon }\right\}$$

where the constitutive matrix is expressed as [35];

$$\left[Q\right]=\left[\begin{array}{cccccc} Q_{11} & Q_{12} & Q_{13} & 0 & 0 & 0\\ Q_{21} & Q_{22} & Q_{23} & 0 & 0 & 0\\ Q_{31} & Q_{32} & Q_{33} & 0 & 0 & 0\\ 0 & 0 & 0 & Q_{44} & 0 & 0\\ 0 & 0 & 0 & 0 & Q_{55} & 0\\ 0 & 0 & 0 & 0 & 0 & Q_{66} \end{array}\right]$$

Here,

(20) $$\begin{array}{l} Q_{11}=Q_{22}=Q_{33}=\frac{E(z)(1-\nu^{2})}{\left(1-3\nu^{2}-2\nu^{3}\right)},\\ Q_{12}=Q_{13}=Q_{23}=\frac{E(z)\nu (1+\nu )}{\left(1-3\nu^{2}-2\nu^{3}\right)},\\ Q_{44}=Q_{55}=Q_{66}=\frac{E(z)}{2(1+\nu )} \end{array}$$

### 3.5. Buckling Analysis

The strain energy of the plate may be written as

(21) $$U=\frac{1}{2}{∭\left\{\bar{\varepsilon }\right\}}^{T}\left\{\sigma \right\}dxdydz$$

By putting the value of Equation (19) in the above Equation (21), we obtain

(22) $$U=\frac{1}{2}{\iint \left\{\bar{\varepsilon }\right\}}^{T}\left[Q\right]\left\{\bar{\varepsilon }\right\}dxdy=\frac{1}{2}{\iint \left\{\varepsilon \right\}}^{T}\left[D\right]\left\{\varepsilon \right\}dxdy$$

where $\left[D\right]=\int {\left[T(z)\right]}^{T}\left[Q\right]\left[T(z)\right]dz$.

The global stiffness matrix of the multiscale composite plate is obtained by equating the total energy of the system to zero.

(23) $$\left[K\right]=\iint {\left[B\right]}^{T}\left[D\right]\left[B\right]dxdy$$

To derive the membrane stiffness matrix $\left[K_{m}\right]$, the membrane strain associated with the deflection can be calculated as [36] follows:

(24) $$\left\{\varepsilon_{m}\right\}=\left\{\begin{array}{l} \frac{1}{2}{\left(\frac{\partial w}{\partial x}\right)}^{2}+\frac{1}{2}{\left(\frac{\partial u}{\partial x}\right)}^{2}+\frac{1}{2}{\left(\frac{\partial v}{\partial x}\right)}^{2}\\ \frac{1}{2}{\left(\frac{\partial w}{\partial y}\right)}^{2}+\frac{1}{2}{\left(\frac{\partial u}{\partial y}\right)}^{2}+\frac{1}{2}{\left(\frac{\partial v}{\partial y}\right)}^{2}\\ \left(\frac{\partial w}{\partial x}\right)\left(\frac{\partial w}{\partial y}\right)+\left(\frac{\partial u}{\partial x}\right)\left(\frac{\partial u}{\partial y}\right)+\left(\frac{\partial u}{\partial x}\right)\left(\frac{\partial v}{\partial y}\right) \end{array}\right\}$$

Or it can be written as

(25) $$\left\{\varepsilon_{m}\right\}=\frac{1}{2}\left[A_{m}\right]\left\{\theta \right\}$$

where $\left[A_{m}\right]=\left[\begin{array}{cccccc} \left(\frac{\partial w}{\partial x}\right) & 0 & \left(\frac{\partial u}{\partial x}\right) & 0 & \left(\frac{\partial v}{\partial x}\right) & 0\\ 0 & \left(\frac{\partial w}{\partial y}\right) & 0 & \left(\frac{\partial u}{\partial y}\right) & 0 & \left(\frac{\partial v}{\partial y}\right)\\ \left(\frac{\partial w}{\partial y}\right) & \left(\frac{\partial w}{\partial x}\right) & \left(\frac{\partial u}{\partial y}\right) & \left(\frac{\partial w}{\partial x}\right) & \left(\frac{\partial v}{\partial y}\right) & \left(\frac{\partial v}{\partial x}\right) \end{array}\right]$, and $\left\{\theta \right\}=\left\{\begin{array}{c} \frac{\partial w}{\partial x}\\ \frac{\partial w}{\partial y}\\ \frac{\partial u}{\partial x}\\ \frac{\partial u}{\partial y}\\ \frac{\partial v}{\partial x}\\ \frac{\partial v}{\partial y} \end{array}\right\}$.

The matrix $\left\{\theta \right\}$ and strain vector $\left\{{\bar{\varepsilon }}_{m}\right\}$ can be related as

(26) $$\left\{\theta \right\}={\left[T_{m}\right]}_{6X26}{\left\{{\bar{\varepsilon }}_{m}\right\}}_{26X1}$$

where

$${\left[T_{m}\right]}_{6X26}=\left[\begin{array}{cccccccccccccccccccccccccc} 0 & 0 & 0 & 0 & 1 & 0 & 0 & 0 & 0 & 0 & z & 0 & 0 & 0 & 0 & 0 & z^{2} & 0 & 0 & 0 & 0 & 0 & 0 & 0 & 0 & 0\\ 0 & 0 & 0 & 0 & 0 & 1 & 0 & 0 & 0 & 0 & 0 & z & 0 & 0 & 0 & 0 & 0 & z^{2} & 0 & 0 & 0 & 0 & 0 & 0 & 0 & 0\\ 1 & 0 & 0 & 0 & 0 & 0 & f_{3}(z) & 0 & 0 & 0 & 0 & 0 & f_{4}(z) & 0 & 0 & 0 & 0 & 0 & f_{5}(z) & 0 & 0 & 0 & f_{6}(z) & 0 & 0 & 0\\ 0 & 1 & 0 & 0 & 0 & 0 & 0 & f_{3}(z) & 0 & 0 & 0 & 0 & 0 & f_{4}(z) & 0 & 0 & 0 & 0 & 0 & f_{5}(z) & 0 & 0 & 0 & f_{6}(z) & 0 & 0\\ 0 & 0 & 1 & 0 & 0 & 0 & 0 & 0 & f_{3}(z) & 0 & 0 & 0 & 0 & 0 & f_{4}(z) & 0 & 0 & 0 & 0 & 0 & f_{5}(z) & 0 & 0 & 0 & f_{6}(z) & 0\\ 0 & 0 & 0 & 1 & 0 & 0 & 0 & 0 & 0 & f_{3}(z) & 0 & 0 & 0 & 0 & 0 & f_{4}(z) & 0 & 0 & 0 & 0 & 0 & f_{5}(z) & 0 & 0 & 0 & f_{6}(z) \end{array}\right]$$

Here, $f_{3}(z)=\left(z-\frac{4z^{3}}{3h^{2}}\right)$, $f_{4}(z)=\left(-\frac{z^{2}}{2}\right)$, $f_{5}(z)=\left(-\frac{4z^{3}}{3h^{2}}\right)$, and $f_{6}(z)=\left(-\frac{z^{3}}{3}\right)$.

By using Equation (26) and the strain displacement relationship, the stress stiffness matrix $\left[K_{m}\right]$ can be written as follows:

(27) $$\left[K_{m}\right]=\iint {\left[B\right]}^{T}\left[I\right]\left[B\right]dxdy$$

where $\left[I\right]=\int {\left[T_{m}(z)\right]}^{T}\left[S\right]\left[T_{m}(z)\right]dz$ and the stress matrix $\left[S\right]$ in terms of plane stress $N_{x}$, $N_{y}$, and $N_{xy}$ can be expressed as follows:

$$\left[S\right]=\left[\begin{array}{cccccc} N_{x} & N_{xy} & 0 & 0 & 0 & 0\\ N_{xy} & N_{y} & 0 & 0 & 0 & 0\\ 0 & 0 & N_{x} & N_{xy} & 0 & 0\\ 0 & 0 & N_{xy} & N_{y} & 0 & 0\\ 0 & 0 & 0 & 0 & N_{x} & N_{xy}\\ 0 & 0 & 0 & 0 & N_{xy} & N_{y} \end{array}\right]$$

The governing equation for calculating the critical buckling is expressed as follows:

(28) $$\left\{\left[K\right]-\lambda \left[K_{m}\right]\right\}\left\{\delta \right\}=0$$

### 3.6. Computation of the Critical Buckling Load

In this analysis, the governing equation for buckling analysis $\left[K\right]\left\{\delta \right\}=\lambda \left[K_{m}\right]\left\{\delta \right\}$ was solved by the simultaneous iteration technique of Corr and Jennings [1] for the computation of eigenvalues and eigenvectors. In this method, $\left[K\right]$ is positive definite and can be decomposed into Cholesky factors as

(29) $$\left[K\right]=\left[L\right]{\left[T\right]}^{T}$$

(30) $$\left\{{\left[L\right]}^{-1}\left[K_{m}\right]{\left[L\right]}^{-T}\right\}{\left[L\right]}^{T}\left\{\delta \right\}=\frac{1}{\lambda }{\left[L\right]}^{T}\left\{\delta \right\}$$

The governing equation for buckling analysis characterizes the standard eigenvalue problem, and these have been solved to extract the eigenvalues and the eigenvectors. In this equation, 1/λ is the eigenvalue. Therefore, the eigenvalue corresponding to the lowest buckling loads is obtained using the simultaneous iteration technique. The methodology is explained as follows:

1. Set a trial vector $\left[U\right]$ and ortho-normalize.
2. Back substitute $\left[L\right]\left[X\right]=\left[U\right]$
3. Multiply $\left[Y\right]=\left[M\right]\left[X\right]$ or $\left[Y\right]=\left[K_{m}\right]\left[X\right]$
4. Forward substitute ${\left[L\right]}^{T}\left[V\right]=\left[Y\right]$
5. Form ${\left[B\right]}^{}{\left[U\right]}^{T}=\left[V\right]$
6. Construct $\left[L\right]$ so that $t_{ji}=1$ and $t_{ij}=\frac{-2b_{ij}}{\left[b_{ii}-b_{ij}+s{\left(b_{ii}-b_{ij}\right)}^{2}\right]}$, where S is the sign of $\left(b_{ii}-b_{ij}\right)$
7. Multiply $\left[W\right]=\left[V\right]\left[T\right]$

The numerical results are calculated in the form of non-dimensional critical buckling as shown below:

(31) $$N^{*}=N_{cr}\frac{a^{2}}{E_{m}\;h^{3}}$$

**Figure 4:**
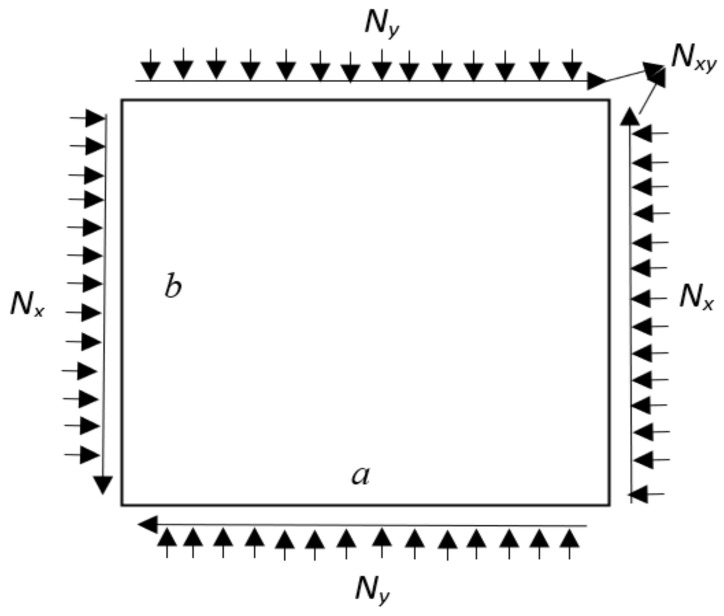
Rectangular plate subjected to bi-axial compressive load and in-plane shear load.

The rectangular plates shown in Figure 4 are subjected to in-plane loading in two different directions. In the given Figure 4 $N_{x}$, $N_{y}$, and $N_{xy}$ are the in-plane axial loading and shear loading, where $N_{x}=-p_{1}N_{cr}$, $N_{y}=-p_{2}N_{cr}$, and $N_{xy}=0$.

The different loading conditions are

Uniaxial compression: $p_{1}=-1$ and $p_{2}=0$
Biaxial compression: $p_{1}=-1$ and $p_{2}=-1$

Boundary conditions are

- SSSS:
  *8.* $\begin{array}{l} v_{0}=w_{0}=\omega_{1}=\alpha_{1}=\eta_{3}=\beta_{1}=\psi_{2}=0\;\;at\;x=0,a\\ u_{0}=w_{0}=\omega_{2}=\alpha_{1}=\eta_{3}=\beta_{2}=\psi_{1}=0\;\;at\;y=0,b \end{array}$
- CCCC:
  $\begin{array}{l} v_{0}=w_{0}=\omega_{1}=\omega_{2}=\omega_{3}=\alpha_{1}=\alpha_{2}=\eta_{3}=\beta_{1}=\beta_{2}=\psi_{1}=\psi_{2}=0,\;at\;x=0,\;a\\ u_{0}=w_{0}=\omega_{1}=\omega_{2}=\omega_{3}=\alpha_{1}=\alpha_{2}=\eta_{3}=\beta_{1}=\beta_{2}=\psi_{1}=\psi_{2}=0,\;at\;y=0,b \end{array}$
- CFCF:
  $\begin{array}{l} v_{0}=w_{0}=\omega_{1}=\omega_{2}=\omega_{3}=\alpha_{1}=\alpha_{2}=\eta_{3}=\beta_{1}=\beta_{2}=\psi_{1}=\psi_{2}=0,\;\;at\;x=0\;\\ v_{0}=w_{0}=\omega_{1}=\omega_{2}=\omega_{3}=\alpha_{1}=\alpha_{2}=\eta_{3}=\beta_{1}=\beta_{2}=\psi_{1}=\psi_{2}=0,at\;y=0\\ u_{0}=v_{0}=w_{0}=\omega_{1}=\omega_{2}=\omega_{3}=\alpha_{1}=\alpha_{2}=\eta_{3}=\beta_{1}=\beta_{2}=\psi_{1}=\psi_{2}\neq 0,\;\;at\;x=a\;and\;y=b \end{array}$
- SSCC:
  $\begin{array}{l} v_{0}=w_{0}=\omega_{1}=\alpha_{1}=\eta_{3}=\beta_{1}=\psi_{2}=0\;\;at\;x=0,a\\ u_{0}=w_{0}=\omega_{1}=\omega_{2}=\omega_{3}=\alpha_{1}=\alpha_{2}=\eta_{3}=\beta_{1}=\beta_{2}=\psi_{1}=\psi_{2}=0,\;\;at\;y=0,b\; \end{array}$
- SCSC:
  $\begin{array}{l} u_{0}=w_{0}=\omega_{2}=\alpha_{1}=\eta_{3}=\beta_{2}=\psi_{1}=0,\;\;at\;x=0\;and\;y=0\\ v_{0}=w_{0}=\omega_{1}=\omega_{2}=\omega_{3}=\alpha_{1}=\alpha_{2}=\eta_{3}=\beta_{1}=\beta_{2}=\psi_{1}=\psi_{2}=0,\;\;at\;x=a\;\\ u_{0}=w_{0}=\omega_{1}=\omega_{2}=\omega_{3}=\alpha_{1}=\alpha_{2}=\eta_{3}=\beta_{1}=\beta_{2}=\psi_{1}=\psi_{2}=0,\;at\;y=b \end{array}$

## 4. Numerical Results

To calculate the critical buckling load, the eigenvalue problem was determined. Various numerical results were used to obtain the mechanical buckling behavior of CNT-reinforced multiscale FGM rectangular plates using the proposed 9-noded isoparametric elements. The finite element code was developed in Matlab to perform the numerical simulation. The numerical values were calculated for 3 × 3 gauss integration points. The material components adopted in this study are listed in Table 1.

### 4.1. Comparison and Convergence

To determine the best suitable mesh size for the present numerical analysis, the given plate was divided into various mesh sizes in the ×- and y-directions. This convergence study was carried out for different volume fractions of ceramic fiber and with a side-to-thickness ratio a/h = 10, as shown in Table 2. The non-dimensional critical buckling load was determined for mesh sizes varying from 2 × 2 to 6 × 6. It was observed that the critical buckling load converged for the mesh size 5 × 5. Therefore, a 5 × 5 mesh size was adopted for the complete numerical analysis.

**Figure 5:**
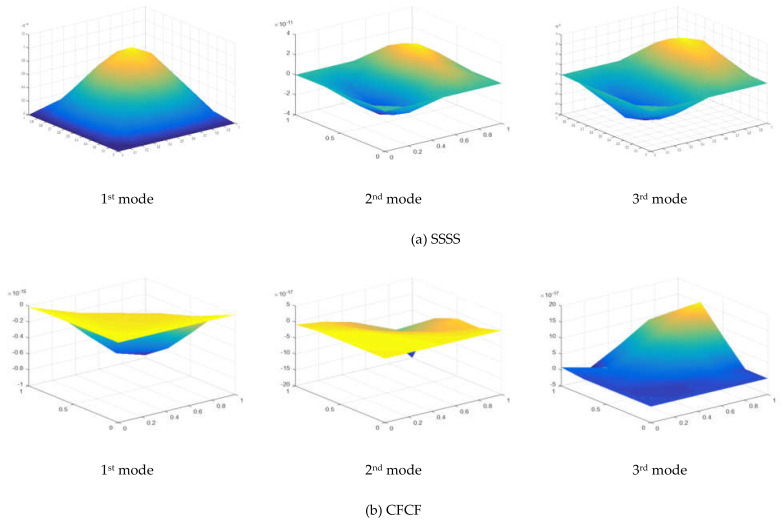
Mode shape for the square plate under biaxial compressive load.

**Figure 6:**
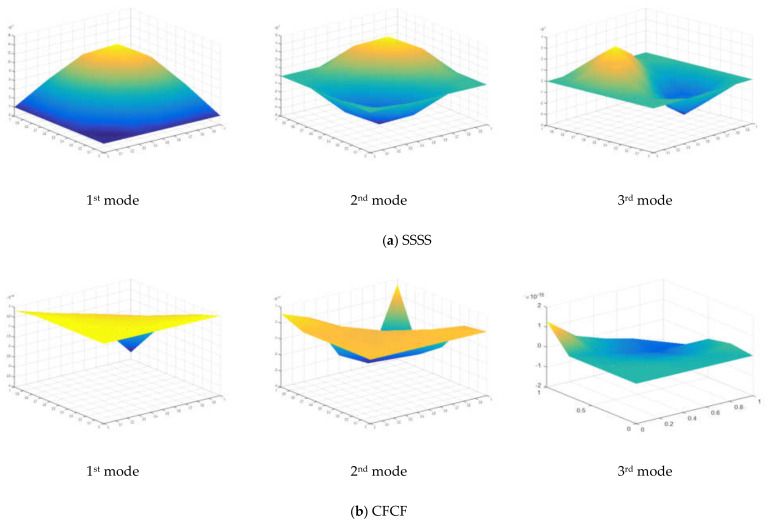
Mode shape for the square plate under shear load.

To validate the present MTSDT theory, the non-dimensional critical buckling load was calculated for a different side-to-thickness ratio of simply supported square plates under uniaxial and biaxial compressive loadings. The numerical values in Table 3 represent the critical buckling load for the Al/Al_2_O_3_ plate with 0% weight fraction of CNT reinforcement. The presented numerical results were compared with a previous numerical study [37] and are in good agreement with the reference. The mode shape of a simply supported plate for the first three nodes is presented in Figure 5 and Figure 6 for *n* = 0 and *n* = 1, respectively.

### 4.2. Effect of Boundary Conditions on Uniaxial and Biaxial Compression

The variation of the non-dimensional critical buckling load for various boundary conditions is represented in Table 4. The numerical values were calculated for 0%, 2.5%, and 5% weight fraction of CNT reinforcement under uniaxial and biaxial loading. From Table 4, the maximum value of the critical buckling load was obtained by clamped (CCCC) boundary conditions, whereas the CFCF boundary condition yielded the minimum value of the critical buckling load. The CCCC boundary condition indicated that the plates were fixed on all four sides, and the CFCF boundary condition indicated that the plates were fixed and free on adjacent sides. In the case of a 0% weight fraction of CNT, approximately (80–85)% difference in the critical buckling load was observed between the CCCC and CFCF boundary conditions, and a (60–63)% difference was observed between the CCCC and SSSS boundary conditions. However, if we assume a 5% weight fraction of CNT in the mixture, slightly less difference was observed between these boundary conditions. From Table 4, it was also observed that for all boundary conditions, the plate had a higher critical buckling load under uniaxial compression than under biaxial compression.

The first three mode shapes of the plate under biaxial compressive and shear loading are presented in Figure 5 and Figure 6, respectively. The mode shapes were drawn for simply supported and clamped-free boundary conditions. As seen from the mode shape of the plate, the essential boundary conditions were satisfied at the supports.

### 4.3. Effect of CNT and Volume Fraction Index (n) on the Critical Buckling Load

**Figure 7:**
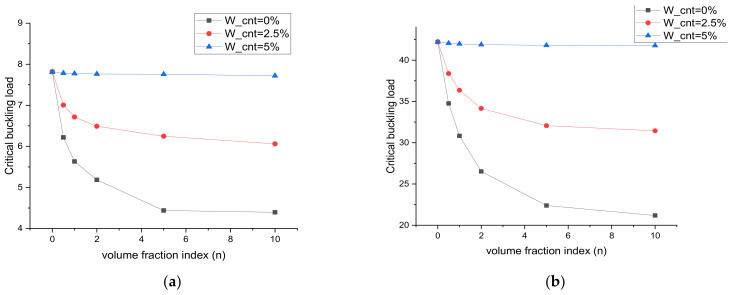

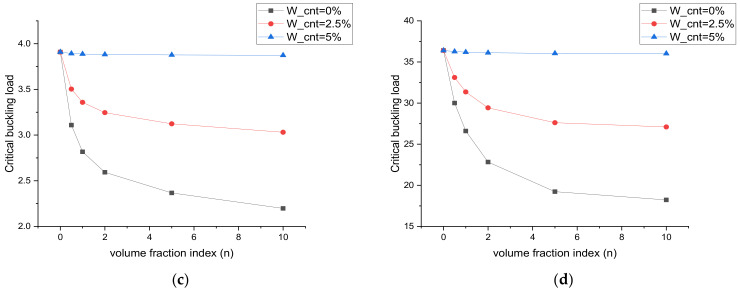
Variation in the critical buckling load for different fractions of CNT and fibers. (**a**) SSSS, (**b**) CCCC, (**c**) SSSS, (**d**) CCCC.

The numerical results for the critical buckling load at a different weight fraction of SWCNT and MWCNT are presented in Table 5 and Table 6 under uniaxial and biaxial compressive loading, respectively. All numerical results were obtained for the 1st six modes, and plates were restrained with a simple supported condition. In this case, the Al/ZrO2 plate was assumed to be reinforced with SWCNT and MWCNT at 0%, 2.5%, and 5% weight fractions. From these tables, it was observed that SWCNT performed better than MWCNT under uniaxial and biaxial compression. At a 5% weight fraction of the CNT, SWCNT had a 17% higher critical buckling load for *n* = 0.5 and 37% higher critical buckling load for *n* = 10 than MWCNT. This happened because of the magnitude of the elastic properties of the SWCNT and MWCNT. Since at *n* = 0 only ZrO_2_ fibers were present, no difference was observed. From *n* = 0.5 to 10, the proportion of ZrO_2_ started to decrease and the proportion of the nanocomposite started to increase. Due to this increase in the nanocomposite proportion, a greater difference was observed at *n* = 10. By increasing the volume fraction index from *n* = 0 to *n* = 10, the amount of fiber in the mixture decreased, which led to a decrease in the stiffness of the plate. Therefore, the critical buckling load decreased as the volume fraction index increased. By increasing the weight fraction of the CNT up to 5%, the critical buckling load increased by 43% in the SWCNT case and 13% in the MWCNT case because of the stiffness of the plate increased by increasing the amount of CNT in the mixture. Figure 7 shows plots for freely supported and clamped boundary conditions for different SWCNT fractions and fiber volumes. At W_cnt = 5%, the plate had an approximately equal critical buckling load from *n* = 0 to *n* = 10. The plate with a multiscale phase behaved similar to the plate with only ceramic fiber at W_cnt = 5%. At W_cnt = 0% and 2.5%, the critical buckling load decreased with an increase in the volume fraction index (*n*), but a greater decline was observed at the 0% weight fraction of the CNT.

### 4.4. Effect of the Side-to-Thickness Ratio (a/h) and Aspect Ratio (b/a) of the Plates

**Figure 8:**
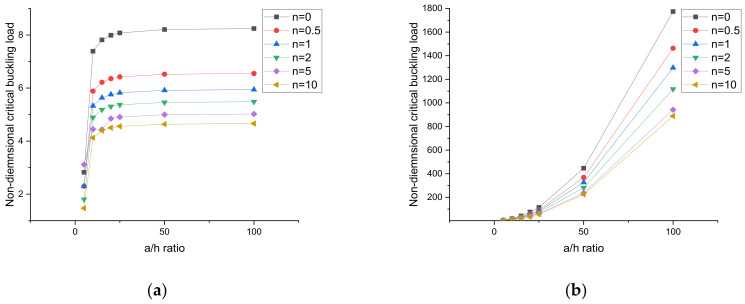
Variation of the buckling load under uniaxial compression. (**a**) SSSS, (**b**) CCCC.

**Figure 9:**
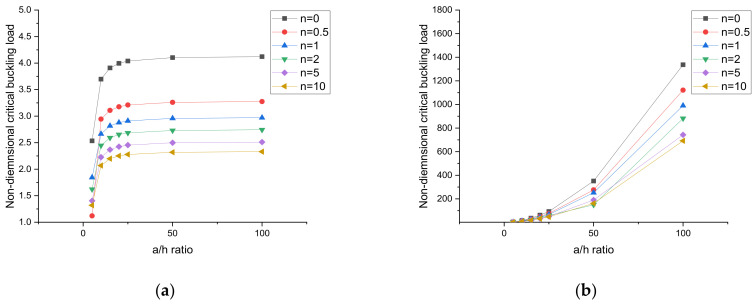
Variation of the buckling load under biaxial compression. (**a**) SSSS, (**b**) CCCC.

The effect of the side-to-thickness ratio of the plate is presented in Figure 8 and Figure 9. Figures are plotted for simply supported and clamped boundary conditions. In this case, all values were calculated for different volume fraction index (*n*) and 0% weight fraction values of the CNT. From Figure 8 and Figure 9, it can be observed that under ceramic-rich conditions, i.e., *n* = 0, the plate had the maximum critical buckling load, and in the case of nanocomposite-rich conditions, i.e., *n* = 10, the plate had the minimum critical buckling load. Figure 8 and Figure 9 present the uniaxial compression and biaxial compression, respectively. Under uniaxial and biaxial compression, the critical buckling load for simply supported plates increased by increasing the a/h ratio up to 20; after that, no significant change in the critical buckling load was observed. This is because in a simply supported plate, the stiffness of the plate increases to only a/h = 20, and the same variation was observed by Reddy et al. [37]. In the case of a clamped supported plate, the critical buckling load increased to a/h = 100 because under clamped support conditions, the stiffness of the plate increased to a/h = 100.

**Figure 10:**
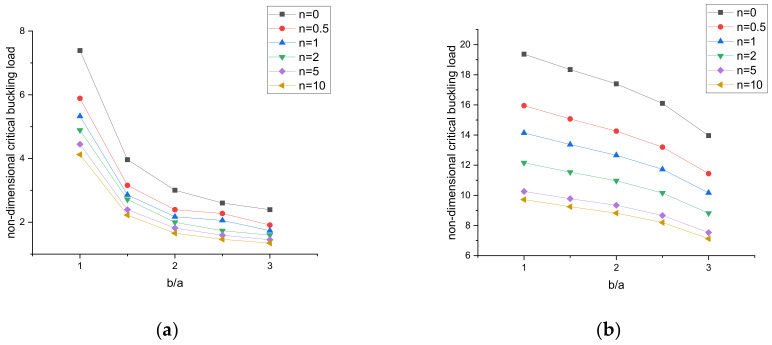
Variation in the buckling load under uniaxial compression. (**a**) SSSS, (**b**) CCCC.

**Figure 11:**
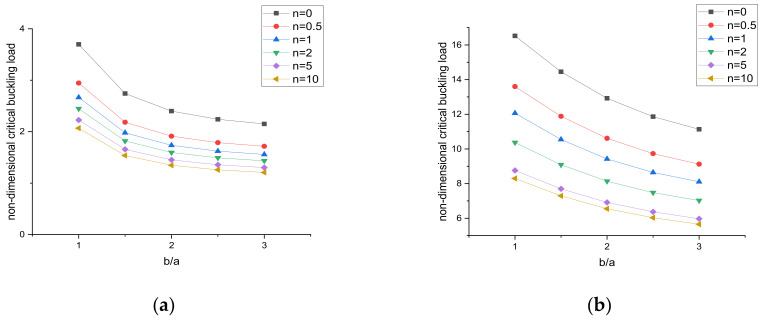
Variation in the buckling load under biaxial compression. (**a**) SSSS, (**b**) CCCC.

The variation of the critical buckling load with the aspect ratio of the plate can be seen in Figure 10 and Figure 11. For the given simply supported and clamped boundary conditions, numerical results were obtained for a constant side-to-thickness ratio of the plate, i.e., a/h = 10. In Figure 10 and Figure 11, it is observed that the critical buckling load increases by increasing the b/a ratio of the plate for both types of loading conditions.

### 4.5. Critical Buckling Load for Various Types of Plates

The variation in the critical buckling load for various types of plates by considering the different volume fraction indexes is presented in Table 7 and Table 8. All numerical values were calculated for the critical buckling load for the 1st six modes for simply supported plates. A plate made of a different type of metal and ceramic fibers behaves differently under uniaxial and biaxial compression. Here, we assumed that the 0% weight fraction of the CNT was used as a reinforcement. In the case of the Al/Al_2_O_3_ plate, the critical buckling load increased by (60–74)% by increasing the volume fraction ratio under uniaxial and biaxial compression. In the case of the Ti-6Al-4V/ZrO_2_ plate, the value for the critical buckling load increased by only (23–28)% by increasing the volume fraction index. Under uniaxial and biaxial compression, the Al/Al_2_O_3_ plate had the highest critical buckling load value among all types of plates made of different metal matrix and fiber components. The differences in the critical buckling load values are due to the different elastic modulus values of the components. In the case of the Al/Al_2_O_3_ plate, the difference in the elastic modulus of the Al matrix and Al_2_O_3_ fiber was much larger. Due to this fact, greater variation in the critical buckling load was observed by increasing the volume fraction index.

### 4.6. Effect of Biaxial and Shear Loading of the Plate

The non-dimensional critical buckling load for a simply supported plate under various in-plane forces is presented in Table 9 and Table 10. Numerical results were calculated for different fiber volume fractions and weight fractions of CNT reinforcement. Table 9 represents the variation in the critical buckling load for the 1st mode under various shear loading and constant biaxial loading values (*N_y_*/*N_x_* = 1). It is noted that by increasing the shear loading from 0 to 2, the non-dimensional critical buckling load decreased by 18% for all fractions of the CNT reinforcement. The reason for this is increased shear loading, reducing the stiffness of the plate.

Table 10 represents the variation in the critical buckling load for various in-plane compressive and shear forces. All values were calculated for a simply supported plate at different weight fractions of the CNT. From Table 10, it can be seen that by increasing the ratio of in-plane compressive forces in the y and × directions, the critical buckling load for all shear loads is reduced. Further, for all uniaxial and biaxial compressive forces, the critical buckling load decreases as the shear load increases. This is because as the compressive and shear loads increase, the buckling resistance of the plate decreases.

## 5. Conclusions

In this paper, an MTSDT mathematical theory was adopted to represent the kinematic field. The in-plane displacement fields integrate the cubic degree of thickness terms and quadratic degree of thickness terms for the out-of-plane displacement field. A nine-noded isoparametric element with 13 unknowns at each node was adopted for the finite element formulation. Effective elastic properties of the multiscale FGM material were predicted by using the Halpin–Tsai equation and the Voigt rule of mixture approach. The effect of various parameters on the critical buckling behavior of a multiscale FGM plate is presented, and the following conclusions were drawn from this numerical analysis:

- The critical buckling load parameter was at a maximum under clamped boundary conditions.
- By increasing the volume fraction index (*n*), the critical buckling is decreased due to less stiffness being obtained at a higher volume fraction index.
- As the weight fraction of CNT increased, the critical buckling load increased because CNT imparted more stiffness to the material.
- The side-to-thickness ratio (a/h) and aspect ratio (b/a) of the plates had a significant impact on the buckling behavior of the plate. Increasing the a/h ratio increased the critical buckling load, and increasing the b/a ratio decreased the critical buckling load.
- Due to the given elastic properties of the Al and Al_2_O_3_, the Al/Al_2_O_3_ plate yielded the maximum value of the critical buckling load among all plates.
- For the same ratio of in-plane compression in the y- and x-direction, the critical buckling load decreased with increases in in-plane shear loading.
- For all values of in-plane shear loading, the critical buckling load decreased with an increase in the ratio of in-plane compression in the y- and x-direction.

It was observed in the present study that CNT fibers and reinforcement play a very important role in the buckling response of a plate structure. The results presented in this study are new for the buckling behavior of multiscale FGM plates. Therefore, it is believed that the results obtained are very useful for the analysis and design of this type of plate structure.

## Figures and Tables

**Table 1:** Material properties of constituents.

Constituents’ Material	Material Properties	
Matrix [9]	Aluminum (Al)	E_m_ = 70 GPa, ν_m_ = 0.3, ρ_m_ = 2707
	Stainless steel (SUS304)	E_m_ = 207.78 GPa, ν_m_ = 0.3177, ρ_m_ = 8166
	Ti-6Al-4V	E_m_ = 105.7 GPa, ν_m_ = 0.298, ρ_m_ = 4429
Fibre [9]	Zirconia (ZrO_2_)	E_c_ = 151 GPa, ν_c_ = 0.3, ρ_c_ = 3000
	Alumina (Al_2_O_3_)	E_c_ = 380 GPa, ν_c_ = 0.3, ρ_c_ = 3800
	Silicon nitride (Si_3_N_4_)	E_c_ = 322.27 GPa, ν_c_ = 0.24, ρ_c_ = 2370
CNT [32]	MWCNT	E_CNT_ = 400 GPa, l_CNT_ = 50 μm, d_CNT_ = 20 nm, t_CNT_ = 0.34 nm, ρ_CNT_ = 1350 kg/m^3^
	SWCNT	E_CNT_ = 640 GPa, l_CNT_ = 25 μm, d_CNT_ = 1.4 nm, t_CNT_ = 0.34 nm, ρ_CNT_ = 1350 kg/m^3^

**Table 2:** Convergence study of the Al/Al_2_O_3_ plate.

	Mesh Size	Volume Fraction Index (*n*)					
		0	0.5	1	2	5	10
a/h = 10	2 × 2	19.192	13.276	11.016	9.161	7.191	6.017
	3 × 3	18.594	9.698	10.410	8.566	6.676	5.701
	4 × 4	18.354	8.131	10.519	8.397	1.917	5.514
	5 × 5	17.516	12.498	9.268	7.671	6.562	5.607
	6 × 6	17.811	12.487	9.888	7.650	6.022	5.022
	[37]	18.570	12.120	9.330	7.260	6.030	5.450

**Table 3:** Comparison study for the Al/Al_2_O_3_ plate.

	a/h		Volume Fraction (*n*)					
			0	0.5	1	2	5	10
Uniaxial	5	Present Study	16.221	10.897	8.322	5.846	5.320	4.329
		Data in [37]	16.000	10.570	8.146	6.230	4.970	4.440
		% error	1.362	3.001	2.115	6.569	6.579	2.564
		Data in [6]	16.021	10.625	8.225	6.343	5.053	4.481
		% error	1.232	2.492	1.172	8.505	5.017	3.504
	10	Present Study	17.516	12.498	9.268	6.671	6.562	5.607
		Data in [37]	18.540	12.080	9.299	7.210	5.990	5.420
		% error	5.846	3.345	0.334	8.080	8.717	3.335
		Data in [6]	18.579	12.123	9.339	7.263	6.035	5.453
		% error	6.066	3.001	0.767	8.876	8.027	2.750
	20	Present Study	19.606	12.785	9.960	8.371	7.084	5.838
		Data in [37]	19.310	12.530	9.649	7.510	6.320	5.750
		% error	1.510	1.995	3.122	10.286	10.785	1.507
		Data in [6]	19.353	12.567	9.668	7.537	6.345	5.767
		% error	1.291	1.707	2.937	9.962	10.435	1.220
	5	Present Study	8.074	5.323	4.095	3.147	2.505	2.242
		Data in [37]	8.001	5.288	4.073	3.120	2.487	2.221
		% error	0.904	0.658	0.537	0.858	0.719	0.937
		Data in [6]	8.011	5.313	4.112	3.172	2.527	2.240
		% error	0.786	0.193	0.420	0.782	0.858	0.076
Biaxial	10	Present Study	9.074	6.183	4.488	3.522	3.056	2.818
		Data in [37]	9.273	6.045	4.650	3.608	2.998	2.715
		% error	2.193	2.232	3.610	2.442	1.898	3.655
		Data in [6]	9.289	6.062	4.670	3.632	3.018	2.726
		% error	2.373	1.965	4.046	3.109	1.253	3.251
	20	Present Study	9.826	6.349	5.020	4.064	3.327	3.062
		Data in [37]	9.658	6.270	4.821	3.757	3.162	2.876
		% error	1.710	1.244	3.964	7.554	4.959	6.074
		Data in [6]	9.676	6.283	4.834	3.769	3.172	2.883
		% error	1.522	1.033	3.711	7.269	4.647	5.833

**Table 4:** Non-dimensional critical buckling load for different boundary conditions.

	W_cnt	Boundary Conditions	Volume Fraction Index (*n*)					
			0	0.5	1	2	5	10
Uniaxial	0%	SSSS	7.389	5.886	5.328	4.891	4.449	4.125
		CCCC	19.367	15.948	14.141	12.158	10.263	9.718
		CFCF	4.415	3.159	2.495	1.881	1.807	1.466
		SSCC	10.267	6.862	6.134	5.557	4.718	4.505
		SCSC	11.242	7.326	6.149	5.257	4.985	4.616
	2.5%	SSSS	7.389	6.626	6.349	5.892	5.723	5.708
		CCCC	19.367	17.611	16.680	15.667	14.710	14.427
		CFCF	4.415	3.277	2.615	2.457	2.281	2.061
		SSCC	10.267	7.725	7.284	6.778	6.662	6.427
		SCSC	11.242	8.326	7.518	7.322	7.149	6.783
	5%	SSSS	7.389	7.358	7.358	7.339	7.328	7.322
		CCCC	19.367	19.297	19.259	19.219	19.181	19.169
		CFCF	4.415	4.248	4.066	4.065	4.025	4.009
		SSCC	10.267	10.037	9.882	9.767	9.682	9.623
		SCSC	11.242	11.126	10.950	10.730	10.551	10.496
Biaxial	0%	SSSS	3.697	2.945	2.665	2.447	2.226	2.068
		CCCC	16.520	13.602	12.062	10.370	8.758	8.298
		CFCF	2.010	1.083	1.066	1.043	0.662	0.559
		SSCC	4.726	4.226	3.855	3.475	2.495	2.103
		SCSC	5.674	4.936	3.952	3.308	2.418	2.218
	2.5%	SSSS	3.697	3.315	3.176	3.066	2.948	2.861
		CCCC	16.520	15.021	14.228	13.364	12.550	12.311
		CFCF	2.010	1.795	1.412	1.291	1.127	1.087
		SSCC	4.726	4.495	4.158	3.619	3.219	3.019
		SCSC	5.674	5.174	4.512	3.853	3.453	3.223
	5%	SSSS	3.697	3.682	3.676	3.672	3.667	3.663
		CCCC	16.520	16.460	16.428	16.393	16.361	16.351
		CFCF	2.010	1.951	1.831	1.811	1.760	1.760
		SSCC	4.726	4.636	4.596	4.556	4.456	4.404
		SCSC	5.674	5.574	5.494	5.395	5.360	5.355

**Table 5:** Critical buckling load for the 1st six modes (Al/ZrO_2_) under uniaxial load.

	W_cnt	Mode	Volume Fraction Index (*n*)					
			0	0.5	1	2	5	10
SWCNT	0%	1	7.816	6.218	5.633	5.186	4.439	4.395
		2	12.650	9.979	8.929	8.135	4.733	6.986
		3	17.908	14.536	12.994	11.378	7.446	9.308
		4	17.927	14.596	13.002	11.468	9.856	9.426
		5	20.211	16.358	14.638	12.980	10.005	10.675
		6	20.687	16.405	14.682	13.301	11.345	11.367
	2.5%	1	7.816	7.005	6.716	6.490	6.246	6.061
		2	12.650	11.324	10.826	10.435	10.042	9.765
		3	17.908	16.186	15.406	14.599	13.814	13.518
		4	17.927	16.228	15.420	14.634	13.883	13.570
		5	20.211	18.264	17.390	16.530	15.689	15.330
		6	20.687	18.534	17.712	17.052	16.387	15.938
	5%	1	7.816	7.784	7.773	7.764	7.756	7.747
		2	12.650	12.599	12.580	12.564	12.543	12.534
		3	17.908	17.840	17.809	17.778	17.735	17.741
		4	17.927	17.860	17.828	17.795	17.769	17.753
		5	20.211	20.133	20.098	20.064	20.018	20.017
		6	20.687	20.603	20.572	20.545	20.522	20.499
MWCNT	2.5%	1	7.816	6.322	5.777	5.360	4.933	4.612
		2	12.650	10.160	9.189	8.451	7.796	7.353
		3	17.908	14.752	13.312	11.804	10.378	9.861
		4	17.927	14.810	13.320	11.886	10.517	9.970
		5	20.211	16.648	15.038	13.448	11.917	11.285
		6	20.687	16.650	15.057	13.815	12.695	11.971
	5%	1	7.816	6.424	5.918	5.530	5.129	4.827
		2	12.650	10.336	9.441	8.757	8.137	7.713
		3	17.908	14.965	13.624	12.222	10.890	10.403
		4	17.927	15.020	13.632	12.296	11.019	10.504
		5	20.211	16.888	15.387	13.908	12.479	11.885
		6	20.687	16.936	15.464	14.312	13.254	12.563

**Table 6:** Critical buckling load for the 1st six modes (Al/ZrO_2_) under biaxial load.

	W_cnt	Mode	Volume Fraction Index (*n*)					
			0	0.5	2	1	5	10
SWCNT	0%	1	3.909	3.109	2.817	2.593	2.367	2.197
		2	10.252	8.082	7.230	6.588	6.037	5.664
		3	10.287	8.111	7.258	6.613	6.057	5.682
		4	12.900	10.355	9.286	8.327	7.396	6.945
		5	13.570	10.994	9.846	8.731	7.656	7.199
		6	13.624	11.038	9.885	8.765	7.685	7.226
	2.5%	1	3.909	3.503	3.358	3.245	3.123	3.031
		2	10.252	9.176	8.773	8.457	8.141	7.915
		3	10.287	9.208	8.803	8.486	8.168	7.941
		4	12.900	11.613	11.081	10.598	10.113	9.860
		5	13.570	12.254	11.674	11.110	10.557	10.310
		6	13.624	12.303	11.720	11.154	10.598	10.350
	5%	1	3.909	3.892	3.887	3.882	3.877	3.873
		2	10.252	10.211	10.195	10.183	10.169	10.159
		3	10.287	10.245	10.230	10.217	10.203	10.193
		4	12.900	12.849	12.828	12.809	12.789	12.779
		5	13.570	13.518	13.494	13.472	13.450	13.440
		6	13.624	13.571	13.548	13.525	13.503	13.493
MWCNT	2.5%	1	3.909	3.161	2.889	2.680	2.467	2.306
		2	10.252	8.229	7.441	6.846	6.321	5.961
		3	10.287	8.259	7.469	6.871	6.342	5.981
		4	12.900	10.522	9.525	8.631	7.757	7.329
		5	13.570	11.159	10.087	9.045	8.039	7.607
		6	13.624	11.203	10.127	9.081	8.070	7.636
	5%	1	3.909	3.212	2.959	2.765	2.565	2.414
		2	10.252	8.373	7.646	7.095	6.597	6.253
		3	10.287	8.403	7.674	7.120	6.619	6.273
		4	12.900	10.685	9.760	8.927	8.111	7.705
		5	13.570	11.321	10.323	9.354	8.414	8.008
		6	13.624	11.366	10.364	9.391	8.447	8.039

**Table 7:** Critical buckling load for the 1st six modes for various types of plates under uniaxial load.

	Mode	Volume Fraction Index (*n*)					
		0	0.5	1	2	5	10
Al/Al_2_O_3_	1	13.278	12.804	10.410	8.566	6.676	5.701
	2	18.593	15.237	12.280	9.373	6.918	5.889
	3	21.668	16.931	13.787	10.492	7.404	6.294
	4	23.838	19.236	15.538	11.890	8.563	7.254
	5	27.250	19.923	15.771	12.083	9.336	8.262
	6	29.562	28.436	22.198	17.197	11.512	9.704
Al/ZrO_2_	1	7.816	6.218	5.633	5.186	4.439	4.395
	2	12.650	9.979	8.929	8.135	4.733	6.986
	3	17.908	14.536	12.994	11.378	7.446	9.308
	4	17.927	14.596	13.002	11.468	9.856	9.426
	5	20.211	16.358	14.638	12.980	10.005	10.675
	6	20.687	16.405	14.682	13.301	11.345	11.367
Ti-6Al-4V/ZrO_2_	1	4.865	4.360	4.139	3.981	3.812	3.688
	2	5.739	5.097	4.833	4.573	4.317	4.207
	3	6.427	5.606	5.335	5.041	4.755	4.634
	4	6.946	6.540	6.080	5.750	5.426	5.286
	5	7.729	6.877	6.553	6.260	5.974	5.801
	6	10.933	9.715	9.184	8.597	8.040	7.862
SUS304/Si_3_N_4_	1	4.671	4.409	4.202	3.982	3.822	3.021
	2	5.413	5.068	4.731	4.403	4.263	5.405
	3	5.990	5.608	5.228	4.858	4.704	6.184
	4	6.840	6.406	5.978	5.559	5.379	6.853
	5	7.433	6.971	6.586	6.219	5.998	7.782
	6	10.267	9.565	8.815	8.102	7.875	8.571

**Table 8:** Critical buckling load for the 1st six modes for various types of plates under biaxial load.

	Mode	Volume Fraction Index (*n*)					
		0	0.5	1	2	5	10
Al/Al_2_O_3_	1	9.303	6.359	5.206	4.285	3.463	2.949
	2	18.476	13.130	10.677	8.189	5.842	4.949
	3	18.576	13.168	10.691	8.202	5.843	4.960
	4	18.639	13.251	10.778	8.263	5.885	4.983
	5	19.858	13.707	10.884	8.418	6.244	5.354
	6	20.029	13.826	10.982	8.500	6.301	5.399
Al/ZrO_2_	1	3.909	3.109	2.817	2.593	2.367	2.197
	2	10.252	8.082	7.230	6.588	6.037	5.664
	3	10.287	8.111	7.258	6.613	6.057	5.682
	4	12.900	10.355	9.286	8.327	7.396	6.945
	5	13.570	10.994	9.846	8.731	7.656	7.199
	6	13.624	11.038	9.885	8.765	7.685	7.226
Ti-6Al-4V/ZrO_2_	1	2.445	2.170	2.071	1.992	1.907	1.845
	2	4.861	4.352	4.126	3.904	3.686	3.590
	3	4.889	4.376	4.149	3.926	3.708	3.612
	4	4.904	4.391	4.163	3.938	3.718	3.621
	5	5.226	4.663	4.425	4.205	3.986	3.878
	6	5.271	4.703	4.463	4.241	4.020	3.912
SUS304/Si_3_N_4_	1	2.696	2.337	2.206	2.103	1.993	1.913
	2	5.294	4.637	4.344	4.057	3.775	3.652
	3	5.308	4.648	4.354	4.067	3.786	3.663
	4	5.339	4.678	4.382	4.091	3.806	3.682
	5	5.677	4.952	4.642	4.356	4.072	3.937
	6	5.725	4.994	4.682	4.393	4.107	3.970

**Table 9:** Non-dimensional shear buckling load for the simply supported plate.

W_cnt	N_xy_/N_x_	Volume Fraction Index (*n*)					
		0	0.5	1	2	5	10
0%	0	3.697	2.945	2.665	2.447	2.226	2.068
	0.25	3.678	2.930	2.651	2.434	2.214	2.057
	0.5	3.629	2.891	2.615	2.400	2.183	2.028
	1	3.462	2.758	2.493	2.285	2.077	1.931
	2	3.026	2.410	2.035	1.989	1.805	1.681
2.5%	0	3.697	3.315	3.176	3.066	2.948	2.861
	0.25	3.678	3.298	3.160	3.051	2.933	2.847
	0.5	3.629	3.255	3.118	3.010	2.893	2.808
	1	3.462	3.105	3.105	2.870	2.758	2.678
	2	3.026	2.714	2.598	2.505	2.406	2.337
5%	0	3.697	3.682	3.676	3.672	3.667	3.663
	0.25	3.678	3.663	3.658	3.658	3.649	3.645
	0.5	3.629	3.614	3.609	3.605	3.600	3.596
	1	3.462	3.448	3.443	3.439	3.434	3.431
	2	3.026	3.013	3.009	3.005	3.001	2.998

**Table 10:** Non-dimensional biaxial and shear buckling load for the simply supported plate.

W_cnt	*n*	N_y_/N_x_	N_xy_/N_x_				
			0	0.25	0.5	1	2
0%	1	0	6.037	5.328	5.235	5.020	4.542
		0.25	4.264	4.210	4.073	3.679	2.916
		0.5	3.554	3.522	3.441	3.186	2.627
		1	2.665	2.651	2.615	2.493	2.035
		2	1.776	1.772	1.761	1.720	1.593
	2	0	4.891	3.245	4.446	4.101	2.981
		0.25	3.914	3.863	3.733	3.366	2.916
		0.5	3.262	3.233	3.156	2.918	2.400
		1	2.447	2.434	2.400	2.285	1.989
		2	1.631	1.627	1.616	1.577	1.458
	5	0	4.449	1.704	4.224	3.584	2.981
		0.25	3.561	3.514	3.393	3.054	2.413
		0.5	2.968	2.941	2.869	2.650	2.177
		1	2.226	2.214	2.183	2.077	1.805
		2	1.484	1.480	1.470	1.434	1.325
2.5%	1	0	6.349	6.440	5.769	5.215	2.211
		0.25	5.081	5.018	4.856	4.392	3.488
		0.5	4.235	4.198	4.102	3.803	3.141
		1	3.176	3.160	3.118	3.105	2.598
		2	2.117	2.112	2.099	1.726	1.901
	2	0	5.708	6.440	5.724	2.475	1.032
		0.25	4.905	4.844	4.686	4.235	3.360
		0.5	4.088	4.053	3.959	3.668	3.027
		1	3.066	3.051	3.010	2.870	2.505
		2	2.044	2.039	2.026	1.979	1.833
	5	0	5.892	6.556	5.725	4.733	3.742
		0.25	4.716	4.656	4.503	4.068	3.226
		0.5	3.931	3.896	3.805	3.524	2.907
		1	2.948	2.933	2.893	2.758	2.406
		2	2.044	1.960	1.351	1.902	1.762
5%	1	0	7.358	7.244	6.897	6.068	4.521
		0.25	5.881	5.808	5.621	5.085	4.040
		0.5	4.902	4.859	4.748	4.402	3.638
		1	3.676	3.658	3.609	3.443	3.009
		2	2.450	2.445	2.429	2.373	2.201
	2	0	7.339	5.198	6.917	5.971	4.508
		0.25	5.874	5.801	5.614	5.079	4.035
		0.5	4.896	4.853	4.743	4.397	3.633
		1	3.672	3.658	3.605	3.439	3.005
		2	2.448	2.442	2.426	2.371	2.198
	5	0	7.328	4.049	6.917	1.713	4.504
		0.25	5.866	5.793	5.607	5.072	4.029
		0.5	4.889	4.847	4.736	4.391	3.628
		1	3.667	3.649	3.600	3.434	3.001
		2	2.444	2.438	2.423	2.367	2.195

## Data Availability

Not applicable.

## Nomenclature

$E_{M}$, $\rho_{M}$, and $\nu_{M}$	Young’s modulus, density, and Poisson’s ratio of the metal matrix.
$E_{CN}$, d, t, and l	Young’s modulus, diameter, thickness, and length of CNT
$W_{CN}$, $\nu_{{}_{CN}}$, and $\rho_{CN}$	weight fraction, Poisson’s ratio, and density of CNT.
$E_{MNC}$	Young’s modulus of nanocomposite
E(z) and $E_{C}$	Young’s modulus of final material and Young’s modulus of ceramic fiber
$u_{o}$, $v_{o}$ and $w_{o}$	In-plane and out-of-plane displacement on the midplane.
$\omega_{1}$ and $\omega_{2}$	Rotation of the normal about the midplane on y- and x-axes.
$\eta_{1}$, $\eta_{2}$, $\eta_{3}$, $\rho_{1}$ and $\rho_{2}$	Higher-order terms
$N_{i}$	Shape function of the iso-parametric element at the i^th^ node
${\left\{d\right\}}_{i}$	Unknown displacement at the i^th^ node.
${\left\{x\right\}}_{i}$ ${\left\{y\right\}}_{i}$	Cartesian coordinate of the i^th^ node.
[*K*]	Global stiffness matrix.
${\left\{B\right\}}_{20X13}$	Strain–displacement matrix
λ	Critical buckling load
CNT	Carbon nanotube
FGM	Functionally graded material
SWCNT	Single-wall carbon nanotube
MWCNT	Multi-wall carbon nanotube
HSDT	Higher-order shear deformation theory
FEM	Finite element methods
FSDT	1^st^-order shear deformation theory
TSDT	3^rd^-order shear deformation theory
MTSDT	Modified third-order shear deformation theory
SSSS	Simply supported condition
CCCC	Clamped free boundary condition
CFCF	Clamped-free boundary condition
SCSC	Simply supported-clamped boundary condition
SSCC	Simply supported-free boundary condition
